# A Nomogram Combined Radiomic and Semantic Features as Imaging Biomarker for Classification of Ovarian Cystadenomas

**DOI:** 10.3389/fonc.2020.00895

**Published:** 2020-06-01

**Authors:** Shushu Pan, Zhongxiang Ding, Lexing Zhang, Mei Ruan, Yanna Shan, Meixiang Deng, Peipei Pang, Qijun Shen

**Affiliations:** ^1^Department of Radiology, Affiliated Hangzhou First People's Hospital, Zhejiang University School of Medicine, Hangzhou, China; ^2^Department of Radiology, Women's Hospital School of Medicine Zhejiang University, Hangzhou, China; ^3^Department of Pharmaceuticals Diagnosis, GE Healthcare, Hangzhou, China

**Keywords:** ovarian neoplasms, cystadenoma, algorithm, classification, tomography, x-ray computed

## Abstract

**Objective:** To construct and validate a combined Nomogram model based on radiomic and semantic features to preoperatively classify serous and mucinous pathological types in patients with ovarian cystadenoma.

**Methods:** A total of 103 patients with pathology-confirmed ovarian cystadenoma who underwent CT examination were collected from two institutions. All cases divided into training cohort (*N* = 73) and external validation cohort (*N* = 30). The CT semantic features were identified by two abdominal radiologists. The preprocessed initial CT images were used for CT radiomic features extraction. The LASSO regression were applied to identify optimal radiomic features and construct the Radscore. A Nomogram model was constructed combining the Radscore and the optimal semantic feature. The model performance was evaluated by ROC analysis, calibration curve and decision curve analysis (DCA).

**Result:** Five optimal features were ultimately selected and contributed to the Radscore construction. Unilocular/multilocular identification was significant difference from semantic features. The Nomogram model showed a better performance in both training cohort (AUC = 0.94, 95%CI 0.86–0.98) and external validation cohort (AUC = 0.92, 95%CI 0.76–0.98). The calibration curve and DCA analysis indicated a better accuracy of the Nomogram model for classification than either Radscore or the loculus alone.

**Conclusion:** The Nomogram model combined radiomic and semantic features could be used as imaging biomarker for classification of serous and mucinous types of ovarian cystadenomas.

## Introduction

Epithelial neoplasm of the ovary accounts for 60% of all ovary tumors and can be classified as benign, borderline, or malignant (1). Ovarian cystadenomas are the most common benign epithelial neoplasms. The two most common types of cystadenomas are serous (70%) and mucinous (25%), whereas endometrioid and clear cell cystadenoma are rare (2). The endometrioid and clear cell cystadenoma have radiological features similar to those of serous cystadenoma and their diagnosis is mainly based on histopathological examinations of surgical samples (3). The radiological presentation of cystadenoma can be classified as serous or mucinous (2, 4).

Serous cystadenoma do not have mutations in either KRAS or BRAF and malignant transformation is rare (3). For patients with asymptomatic serous cystadenoma, regular follow-ups without invasive intervention are usually recommended (5). KRAS mutations of mucinous cystadenoma are present in up to 58% of cases, and transformation to borderline or malignant carcinoma is common (6–8). In addition, the mucin within mucinous cystadenoma could cause peritoneal seeding and appendiceal mucocele (9, 10). Decisions regarding the treatment of mucinous cystadenoma need to be made proactively depending on the histologic classification.

Ultrasound (US), Magnetic resonance imaging (MRI), and Computed tomography (CT) are widely used in the visualization and differentiation of ovarian cystadenoma (11, 12). These unique characteristics can be qualitative descriptors, termed semantic features, that describe a tumor's shape and internal structure that are scored by radiologists to characterize lesions, such as size, contour, septa, unilocular/multilocular, mural nodules, texture (2, 13, 14). Semantic features are considered qualitative since they are scored according to the visual assessment of radiologists, which limits the extent of the tumor description to what is observable by the eye (15–17).

Radiomic analysis links quantitative imaging features to clinical findings by using machine-learning and statistics-analysis methods. With high-throughput computing, innumerable quantitative features could be extracted from tomographic images [CT, MR or positron emission tomography (PET)] (18–20). Previous work (21, 22) has suggested that MR radiomic features might be affected by factors such as MRI magnetic strength and scan parameters, resulting in poor reproducibility. CT scan has a relatively uniform protocol and CT Radiomics has been used to evaluate grade and prognosis of multiple types of tumors (18, 19, 23, 24). However, there were sparse studies addressed radiomic analysis to differentiate the types of ovarian cystadenoma.

We hypothesized that CT semantic and radiomic features can identify the associations between the tumor imaging phenotypes and pathophysiology. We aimed to develop and validate a combined Nomogram model that integrates radiomic features derived from contrast-enhanced CT images with semantic features to improve the type assessment of ovarian cystadenoma for personalized precision therapy.

## Materials and Methods

**Figure 1:**
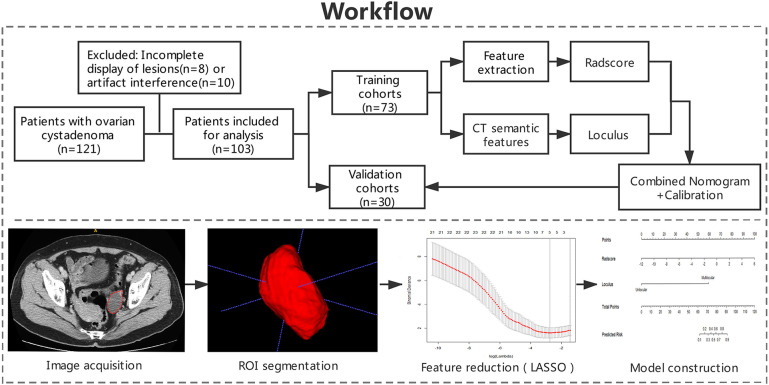
Workflow of the study. Workflow can be divided into four parts: image acquisition, ROI segmentation, feature extraction, and model construction.

This retrospective study was approved by the Medical Ethics Committee of institution I and II and were conducted in accordance with relevant guidelines. Informed consent was waived. The workflow of the analysis is summarized in Figure 1.

### Patients

Patients diagnosed with ovarian cystadenoma with pathological confirmation, who underwent conventional contrast-enhanced CT imaging of abdominal pelvis between December 2017 and June 2019 were retrospectively collected in two institutions. Clinical data were collected by gynecologist including age, lesion location, CA125 level, ascites, pelvic pain, bloating.

The inclusion criteria were as follows: (1) patient with histologic diagnosis of ovarian cystadenomas obtained with surgery in two institutions; (2) preoperative contrast-enhanced CT scans; (3) no chemotherapy or radiation therapy prior to CT scans.

Exclusion criteria were as follows: (1) any artifacts within the scan area that affected the display of lesion; (2) the scan area did not cover the entire lesion.

### CT Examination

All patients underwent an abdominal pelvis contrast-enhanced CT scan preoperatively. Contrast-enhanced CT scan in Institution I was performed on a 16-slice CT (GE Healthcare, Milwaukee, Wisconsin) and institution II was performed on a 64-slice CT (Philips Healthcare, Cleveland, Ohio). Both institutions applied the same imaging protocols. The non-ionic contrast agent Ultravist^®^ (Bayer Schering Pharma, Berlin, Germany) was bolus-injected (1.5 mL/kg) with a high-pressure syringe at 3.0 mL/s. Eighty seconds after contrast medium injection, venous phase contrast-enhanced CT images were acquired. The scan parameters: tube voltage of 120 kVp, a pitch value of 0.99, a matrix of 512 × 512, slice thickness and interval were both 5 mm, and milliamperage was adjusted automatically according to the patient's size (ranged between 220 and 400 mA).

### Imaging Evaluation

CT semantic features were assessed by two abdominal radiologists (both with 20 years of experience) in CT images, who were blind to the pathological and clinical data, including size, lobulated contour, thin wall, septa, and loculus. The unilocular was characterized by only one closed loculus or cavity; multilocular was defined more than or equal to two closed loculi. Thin wall was identified <3 mm (2).

### Image Processing

The contrast-enhanced CT images of enrolled patients were exported in Digital Imaging and Communication in Medicine (DICOM) format in two institutional picture archiving and communication system (PACS). Two radiologists (with 4 years of experience and 14 years of experience) who were blinded to the clinical data, evaluated the contrast-enhanced CT images using ITK-SNAP (Version 3.6) software. Before delineation, gray-level standardization was applied to reduce the gray-level differences caused by the imaging procedure. To avoid false heterogeneity assumption at the lesion edge area, the region of interest (ROI) was delineated manually layer by layer along the pixels on the inner edge of the lesion to eventually show a three-dimensional image of the tumor region (Figure 1). The ROI contours were superimposed to improve the consistency of tumor segmentation. All pixel's gray levels inside the ROI were extracted for analysis.

### Feature Extraction, Radscore Building, and Correlation

A total of 396 radiomic features from ROIs were extracted from preprocessed images using the Artificial Intelligence Kit Version 3.0.1.A (Life sciences, GE Healthcare, US). Six main categories were involved, including histogram, morphology, texture parameters, gray level co-occurrence matrix (GLCM), gray level run-length matrix (GLRLM), and gray level zone size matrix (GLZSM). Features were calculated with the following parameters: window width 400, window level 40, GLCM bin number 50, GLRLM bin number 50, GLZSM bin number 200. ANOVA-KW (The analysis of variance and Kruskal-Wallis test) and single-factor logistic regression analysis were successively carried out for selecting significant features that were highly correlated. By removing the redundancy with correlation coefficient more than 0.90, radiomic features were further optimally elected. In the final step, the least absolute shrinkage and selection operator (LASSO) regression method was applied to identify the most non-redundant and robust features among the 396 radiomic features from the training cohort in order to improve the class separability and optimize the representation of lesion heterogeneity (Figure 1). The binomial deviance in the logistic regression model fitting method was used as the criterion to select the best value of λ (25). The λ value with the least binomial deviance was used for the final LASSO regression by conducting 10-fold cross validation method. Meanwhile, the best value of λ found by 10-fold cross-validation with a maximum area under the curve (AUC) was used for constructing the regression model (26, 27). Details of the procedures for extraction of radiomic features were described in Supplementary Materials.

Radscore which defined by corresponding non-zero coefficients of features selected by LASSO, was created by a linear combination of selected features weighted by their coefficients. Respective Radscore was calculated for each patient.

The Pearson correlation analysis was performed to evaluate the correlation between the loculus and Radscore, the pair-wise Pearson correlation coefficients were calculated.

### Nomogram Building, Calibration, and External Validation

Both the Radscore and optimal semantic feature were integrated by a multivariate logistic regression analysis in the training cohort. Based on this, a Nomogram was constructed for classification of ovarian cystadenoma. The constructed Nomogram model was validated by the external validation cohort using the same process of capability assessment with the ROC analysis and calibration curve. Decision curve analysis (DCA) was carried out to evaluate the clinical value of the three models (Radscore, loculus, and Nomogram model) on the basis of calculating the net benefit for patients at each threshold probability.

### Statistical Analysis

Statistical analysis was conducted by SPSS software (Version 19.0) and R software (Version 3.3.2). Variables of a normal distribution were shown as mean ± *SD*, and variables of a skew distribution were shown as median (Quartile). Statistical group comparisons of data were analyzed by χ^2^ tests and Wilcoxon using rank-sum. *P* < 0.05 were considered statistically significant. The agreement between two radiologists was evaluated using interclass correlation coefficient (ICC) analysis, which was defined as good consistency between 0.75 and 1, fair consistency between 0.4 and 0.75, and poor under 0.4. The correlation and collinearity of radiomic features were evaluated using VIF function. The loculus, Radscore, and Nomogram model were respectively subjected to ROC analysis, by using area under the curve (AUC), sensitivity, specificity, and accuracy to evaluate the classification efficacy. The comparison of ROC curves was performed by Delong's test.

## Results

### Patients Characteristics and Conventional CT Findings

A total of 103 cases with pathologically confirmed ovarian cystadenoma were selected in the final cohort. The 103 cases were divided into a training cohort (*N* = 73) and a validation cohort (*N* = 30) (Figure 1). The serous and mucinous cystadenoma had an even distribution in patient characteristics. No significant difference was found in ovarian cystadenoma clinical characteristics (age, location of lesion, the tumor marker CA125 level, ascites, pelvic pain, bloating) between two groups (Table 1).

**Table 1:** Clinical characteristics of training and validation cohorts.

**Characteristics**	**Training cohorts**		***P*-value**	**Validation cohorts**		***P*-value**
	**Serous**	**Mucinous**		**Serous**	**Mucinous**	
	**(*n* = 34)**	**(*n* = 39)**		**(*n* = 15)**	**(*n* = 15)**	
Age (mean ±*SD*)	47.68 ± 12.76	43.00 ± 10.83	0.451	46.64 ± 15.32	40.92 ± 13.77	0.704
Lesion location (%)			0.370			0.700
Unilateral	22 (65%)	29 (74%)		9 (60%)	11 (73%)	
Bilateral	12 (35%)	10 (26%)		6 (40%)	4 (27%)	
CA125	4 (12%)	6 (15%)	0.742	1 (7%)	2 (13%)	1.000
Ascites	5 (15%)	4 (10%)	0.564	2 (13%)	1 (7%)	1.000
Pelvic pain	12 (35%)	8 (21%)	0.194	5 (33%)	3 (20%)	0.682
Bloating	13 (38%)	9 (23%)	0.204	6 (40%)	3(20%)	0.427

Conventional CT semantic features including lesion size, lobulated contour, thin-wall, septa, loculus (Unilocular/multilocular). Size, lobulated contour, thin-wall, septa were no significant difference between two groups, However, loculus (Unilocular/multilocular) identification was significant difference in both cohorts (*p* < *0.05*). The detailed distribution of CT semantic features in the two groups were summarized in Table 2.

**Table 2:** CT semantic features of training and validation cohorts.

**CT semantic features**	**Training cohorts**		***P*-value**	**Validation cohorts**		***P*-value**
	**Serous**	**Mucinous**		**Serous**	**Mucinous**	
	**(*n* = 34)**	**(*n* = 39)**		**(*n* = 15)**	**(*n* = 15)**	
Size	9.69 ± 4.87	11.52 ± 5. 83	0.250	8.91 ± 5.01	10.81 ± 4.03	0.370
Lobulated contour	7 (21%)	17 (44%)	0.087	4 (27%)	7 (47%)	0.225
Thin wall	34 (100%)	36 (92%)	0.243	15 (100%)	13 (87%)	0.483
Septa	16 (47%)	27 (69%)	0.062	7 (47%)	12 (80%)	0.128
Loculus			0.001^*^			0.009^*^
Unilocular	24 (71%)	7 (18%)		10 (67%)	3 (20%)	
Multilocular	10 (29%)	32 (82%)		5 (33%)	12 (80%)	

* *indicates statistical significance*.

### Reproducibility Analysis

Based on the result of reproducibility analysis by two radiologists, 351 out of 396 (88.6%) radiomic features and all the semantic features had good consistency (ICC ≥ 0.75). The number of features with fair consistency (0.75 > ICC ≥ 0.4) and poor consistency (ICC <0.4) were 25 (6.3%) and 20 (5.1%), respectively. Table 3 showed the ICC value of significant features.

**Table 3:** Reproducibility analysis of significant features.

**Significant features**	**Feature class**	**ICC (95%CI)**
CS_AD,o1_	GLCM	0.905 (0.649~0.975)
C_a90,o7_	GLCM	0.862 (0.488~0.963)
LRHGLE_a0,o7_	GLRLM	0.968 (0.882~0.991)
LRHGLE_a90,o7_	GLRLM	0.923 (0.714~0.979)
LISAE	GLSZM	0.921 (0.705~0.979)
Loculus	CT semantic features	1.000

*GLCM, gray level co-occurrence matrix; GLRLM, gray level run-length matrix; GLSZM, gray level zone size matrix; AD, All Direction; a, angle; o, offset*.

### Radscore Model Building, Correlation, and Validation

A total of 396 radiomic features were extracted using AK software. A significance level of 0.05 was set as the threshold. After dimensionality reduction, which included ANOVA and KW, univariate logistic regression (143 features), remove the redundancy with correlation coefficient more than 0.90 (28 features) and after the LASSO algorithm with a value of λ = 0.001445 and log (λ) = −2.84, five significant radiomic features were identified. The complete details were shown in Supplementary Materials.

To demonstrate the effectiveness of radiomic features model at the individual scale, the quantitative values of radiomic features for each patient regarding the classification of serous and mucinous cystadenoma groups were shown in Table 4, which included ClusterShade_AllDirection_offset1 (CS_AD,o1_), Correlation_angle90_offset7 (C_a90,o7_), Long Run High Gray Level Emphasis_angle0 _offset7 (LRHGLE_a0,o7_), Long Run High Gray Level Emphasis_angle90_offset7 (LRHGLE_a90,o7_), and Low Intensity Small Area Emphasis (LISAE). A Radscore model was further constructed based on five features with respective non-zero coefficients selected through LASSO regression method. There were no collinearity between the five features after being verified by VIF function. The complete details were shown in Supplementary Materials.

(1) $$\begin{array}{l} Radscore\;=-0.009-0.864\times CS_{AD,o1}+1.417\times C_{a90,o7}\\ \;-2.259\times LRHGLE_{a0,o7}+0.1\times LRHGLE_{a90,o7}\\ \;+0.799\times LISAE. \end{array}$$

**Table 4:** Univariate analysis of radiomic features in the training and validation cohorts.

**Radiomic features**	**Training cohorts**		***P*-value**	**Validation cohorts**		***P*-value**
	**Serous (*n* = 34)**	**Mucinous (*n* = 39)**		**Serous (*n* = 15)**	**Mucinous (*n* = 15)**	
CS_AD,o1_	−0.10 (−0.42, 0.56)	−0.47 (−0.62, −0.30)	<0.001^*^	0.22 (−0.36, 0.46)	−0.51 (−0.62, −0.02)	0.037^*^
C_a90,o7_	−0.53 (−0.70, −0.31)	0.07 (−0.47, 0.56)	<0.001^*^	−0.63 (−0.70, −0.46)	0.10 (−0.51, 2.49)	0.021^*^
LRHGLE_a0,o7_	0.16 (−0.26, 1.04)	−0.58 (−0.73, −0.35)	<0.001^*^	−0.12 (−0.34, 1.19)	−0.59 (−0.78, −0.35)	0.026^*^
LRHGLE_a90,o7_	0.34 (−0.37, 0.93)	−0.57 (−0.79, −0.24)	<0.001^*^	0.40 (−0.40, 0.91)	−0.59 (−0.78, 0.26)	0.016^*^
LISAE	−0.32 (−0.77, 0.41)	0.42 (−0.58, 1.14)	0.002*	−0.22 (−0.85, 0.58)	0.42 (−0.58, 1.12)	0.062
Radscore	−1.57 (−3.33, −0.19)	1.65 (0.16, 3.41)	<0.001^*^	−1.44 (−3.16, 0.00)	1.29 (−0.36, 5.58)	0.001^*^

*AD, All Direction; a, angle; o, offset*.

* *indicates statistical significance*.

**Figure 2:**
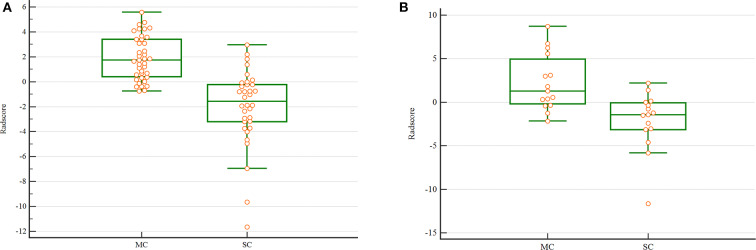
The scatterplot of Radscore. The scatterplot in the training **(A)** and validation **(B)** cohort. (MC. mucinous cystadenoma; SC. serous cystadenoma).

The Radscore had the AUC of the model in training and validation cohorts were 0.88 and 0.84, respectively, which showed higher value of mucinous cystadenoma than serous cystadenoma in both two cohorts (Figure 2).

**Figure 3:**
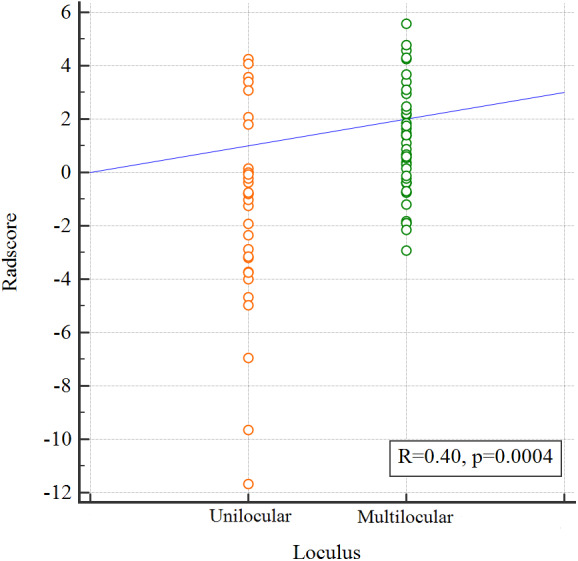
Correlation between the Loculus and the Radscore based on Pearson correlation analysis. The mean absolute correlation coefficient was 0.40.

The pair-wise Pearson correlative analysis revealed that the Radscore was moderate correlated to loculus feature (Figure 3).

### Nomogram Building and Validation

**Figure 4:**
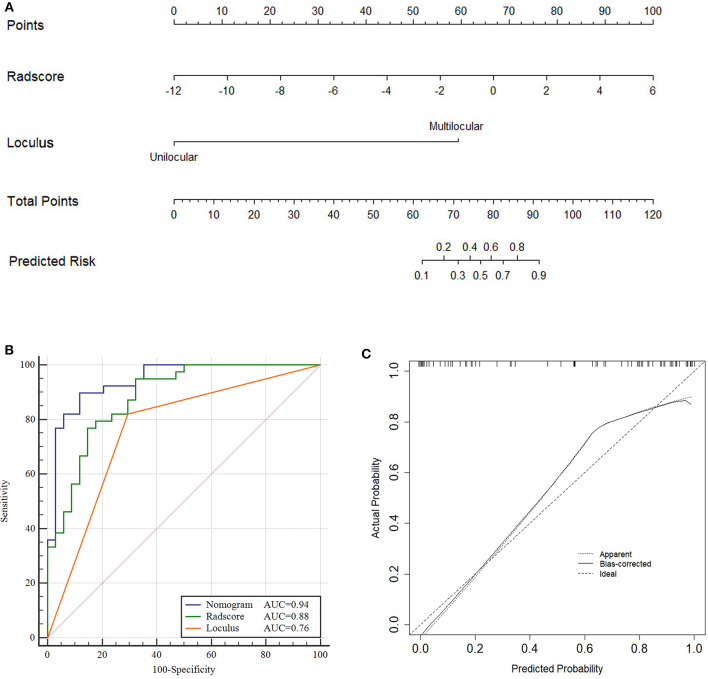
Nomogram, ROC and calibration curves of training cohort. Nomogram **(A)**, To draw an upward vertical line to the “Points” bar to calculate points. Based on the sum, draw a downward vertical line from the “Total Points” line to calculate the probability of classification of ovarian cystadenoma for each patient. For instance, Type serous cystadenomas in a 49-years-old woman with the Radscore value of−1 calculated from the formula, manifesting uniloculus, the corresponding value on the “Points” bar were 62 and 0, respectively. The probability of classification of serous cystadenomas was 88% by drawing a downward vertical line from the value of 62 on “Total Points” bar. ROC curves for the Nomogram, Radscore, and Loculus model **(B)** corresponding calibration curves based on the Nomogram model **(C)** in the training cohort.

The Nomogram based on both Radscore and the loculus was constructed to visualize the results of multivariable logistic regression analysis for classification of ovarian cystadenoma (Figure 4A). *Nomogram* = −0.010 + 0.075 × *Radscore* + 0.346 × *loculus*.

The total points accumulated by the various variables correspond to the predicted probability for a patient (28). The complete details were shown in Figure 4A.

Compared to the Radscore and the loculus alone, the Nomogram model yielded a better performance in the training cohort with a larger AUC value (Table 5 and Figure 4B).The calibration curves in the training cohort demonstrated a high accuracy of the model in the classification capability (Figure 4C).

**Table 5:** Performance of the Loculus, Radscore, and Nomogram models.

**Model**	**Training cohort**				**Validation cohort**			
	**AUC (95%CI)**	**SEN**	**SPEC**	**ACC**	**AUC (95%CI)**	**SEN**	**SPEC**	**ACC**
Radscore	0.88 (0.79–0.95)	0.94	0.68	0.82	0.84 (0.67–0.95)	0.73	0.87	0.80
Loculus	0.76 (0.65–0.85)	0.82	0.71	0.77	0.73 (0.54–0.88)	0.80	0.87	0.73
Nomogram	0.94 (0.86–0.98)	0.90	0.88	0.89	0.92 (0.76–0.98)	0.73	1.00	0.87

*AUC, area under the ROC curve; SEN, sensitivity; SPEC, specificity; ACC, accuracy; AD, All Direction; a, angle; o, offset*.

**Figure 5:**
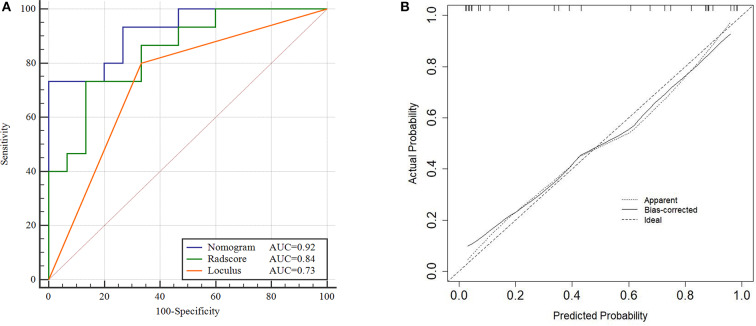
ROC and Calibration curve of validation cohort. Performance of the Nomogram, Radscore and Loculus model on external validation cohort. ROC curve for the three model with an AUC of 0.92, 0.84, and 0.73, respectively **(A)**. Calibration curve of the Nomogram model in the validation cohort **(B)**.

The performance of the Nomogram model was validated using the external dataset collected from the institution II. The Nomogram yielded a favorable AUC value in the validation cohort (Figure 5A). The calibration curves of the proposed Nomogram model based on the validation cohort suggested a favorable classification performance (Figure 5B). Specifically, the Nomogram showed a significant improvement compared to the Radscore and loculus alone in training cohort (*p* < 0.05). The complete details were shown in Supplementary Materials.

**Figure 6:**
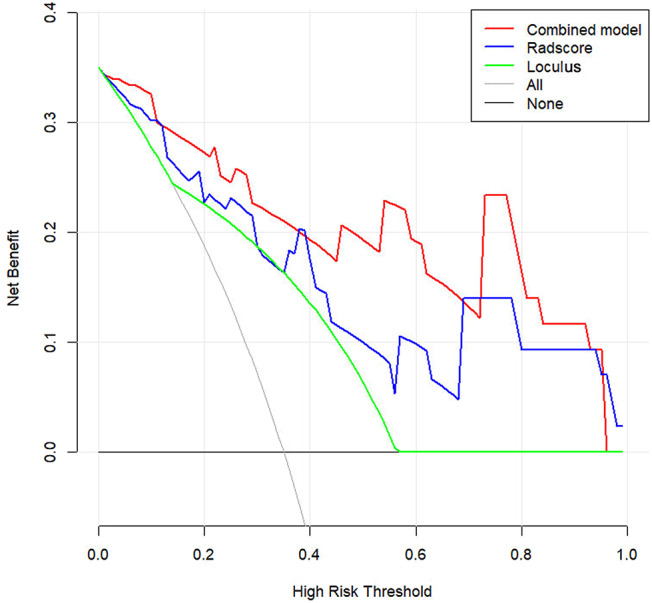
Decision curve analysis (DCA) for the Nomogram model in validation cohort. Compared to other models, the combined Nomogram model, showing the highest area under the curve, is the optimal decision making for maximal net benefit in Classification of Ovarian Cystadenomas.

DCA was conducted to assess the clinical utility of the three models (Figure 6). The Nomogram demonstrated a larger net benefit than did the Radscore and loculus alone, indicating that the Nomogram had the best clinical utility for classification of ovarian cystadenoma in the validation cohort.

## Discussion

In this study, we established and validated a Nomogram model for classification of ovarian cystadenoma, which incorporated five robust radiomic features extracted from contrast-enhanced CT and the semantic features. The Nomogram model achieved a better performance in both training cohort and validation cohort with a larger AUC value than the radiomic model or loculus alone, suggesting the reliability of the improved model for classification of ovarian cystadenoma.

Previous studies have summarized the typical semantic features of serous cystadenoma were often seen as unilocular, thin-walled cystic masses with simple fluid (3). Mucinous cystadenoma were usually seen as multilocular that may be similar or of widely varying size, with liquids of various viscosities (2, 4).Contrast-enhanced CT imaging can differentiate serous from mucinous cystadenoma to a certain extent (3). In this study, the loculus was significant difference between the two groups, multilocular semantic feature might be associated with proteinaceous cellular debris within the fluid, abnormal vasculature, or papillary projections. However, CT semantic features were defined by experienced radiologists, which were still a subjective assessment, and large amounts of quantitative imaging information representing underlying histologic characteristics could not be acquired by visual inspection.

In this study, five optimal quantitative radiomic features were extracted: CS_AD,o1_; C_a90,o7_; LRHGLE_a0,o7_; LRHGLE_a90,o7_; and LISAE. ClusterShade and Correlation are both the gray level co-occurrence matrix (GLCM) parameters. ClusterShade quantitatively analyzes the similarity between objects in the same cluster. Correlation is a value showing the linear dependency of gray level values to their respective voxels in the GLCM (29). Our results suggested that higher (CS_AD,o1_) values and lower (C_a90,o7_) values indicated higher heterogeneity of the lesion. Long Run High Gray Level Emphasis (LRHGLE), which measures image texture smoothness quantitatively, is a parameter for the Gray level run-length matrix (GLRLM) (30). In this study, lower LRHGLE_a0,o7_ and LRHGLE_a90,o7_ values indicating higher heterogeneity of the lesion. LISAE values which measure the uniformity of image texture, is a parameter for the Gray level zone size matrix (GLSZM) (31). In this study, higher LISAE values indicating more heterogeneous textures of the lesion. The Radscore of mucinous group was higher value than that of serous group in both two cohorts, which suggested that the mucinous cystadenoma had greater heterogeneity, as evidenced by the uneven distribution of greyscales and unorganized local texture on the CT images. The Radscore had the AUC of the model in training and validation cohorts were 0.88 and 0.84, respectively.

The radiomic features represented underlying histologic characteristics could not be acquired by visual inspection, meanwhile the loculus of semantic feature represented the morphology of intratumor which could not be extracted by radiomic analysis. Due to the radiomic and semantic features complement each other, the ROC, DCA and calibration analysis results showed the Nomogram model to be more effective and reliable than the radiomic model or semantic features alone. The classification performance of the Nomogram model was validated using an external cohort, demonstrating a strong confirmation of reproducibility by a satisfactory AUC of 0.92. The Nomogram incorporates the five selected radiomic and semantic features which might offer a clinically translatable paradigm easy to implement in the clinical setting.

Although the two radiologists who worked on radiomic analysis differed significantly in their years of experience, the contouring results were relatively consistent (ICC > 0.75). The advantage of a fully quantitative radiomic assessment method is that a wealth of experience in imaging diagnosis is not required. Even a junior physician can accurately delineate tumor at the appropriate window level, and preliminarily classify the type of ovarian cystadenoma.

This study has several limitations. First, we used manual segmentation when delineating the lesion, and therefore we could not completely avoid the interference caused by the partial volume effect. Second, this was a retrospective study with a relatively small dataset in external validation cohort, and further prospective studies are expected to verify the conclusions. Finally, Because of the low incidence of other types of ovarian cystadenomas, they were not included in this study.

## Conclusion

The combined Nomogram integrated radiomic and semantic features can be a reliable and effective model for classification of ovarian cystadenoma, which could serve as a potential marker to classify the type of ovarian cystadenoma and facilitate precision treatment.

## Data Availability Statement

The datasets generated for this study are available on request to the corresponding author.

## Ethics Statement

The studies involving human participants were reviewed and approved by Affiliated Hangzhou First People's Hospital, Zhejiang University School of Medicine; Women's Hospital School of Medicine Zhejiang University. Written informed consent for participation was not required for this study in accordance with the national legislation and the institutional requirements.

## Author Contributions

SP contributed to prepare the manuscript and the statistical analysis. QS put forward the concept of the study, designed the study. ZD reviewed the manuscript. LZ, MR, and YS contributed to the data acquisition, analysis, and interpretation. PP carried out the data analysis. All authors read and approved the final manuscript.

## Conflict of Interest

The authors declare that the research was conducted in the absence of any commercial or financial relationships that could be construed as a potential conflict of interest.
