# Supplementary material for: A Nomogram Combined Radiomic and Semantic Features as Imaging Biomarker for Classification of Ovarian Cystadenomas
Source: Front Oncol. 2020 Jun 1;10:895. doi: 10.3389/fonc.2020.00895 (PMC7277787; doi:10.3389/fonc.2020.00895)
Supplement: Supplementary file 1 [file Data_Sheet_1.doc]

**Supplementary Materials**

**Ⅰ** **Feature extraction**

A total of 396 radiomic features from 103 patients were extracted in this study. According to *P*＜0.05，ANOVA-KW (The analysis of variance and Kruskal-Wallis test), single-factor logistic regression analysis were carried out for selecting significant features that were highly correlated (N=143). By removing the redundancy with correlation coefficient more than 0.90, radiomic features (N=28) were further optimally extracted. In the final step, the LASSO algorithm with a value of λ= 0.001445 and log (λ) = -2.84, five features with non-zero coefficients were finally selected by 10-fold cross validation for ensuring robustness and preventing overfitting.

**Ⅱ The least absolute shrinkage and selection operator (LASSO) method**

The LASSO method is a prevalent high dimensional feature selection method that can be utilized for these radiomic feature data because it can simultaneously perform regularization and variable selection, which can improve both prediction accuracy and interpretation. The radiomic features screening process to select the optimal features for constructing the radiomic signature refers to the procedure that selectively puts a subset group of radiomic features combined into the model to obtain better performance than the one if all the radiomic features were put into the model for fitting.

Complexity adjustment is performed via adjusting a series of parameters to control the complexity of a model during model fitting of LASSO regression in order to avoid overfitting. For a linear model, the complexity is directly related to the number of variables in the model, whereby more variables introduced lead to a higher complexity of model. Adding more variables tends to generate a seemingly “nicer” model while fitting, but it might also increase the risk of overfitting the data. This overfitting usually yields poor results if the validation dataset is used to verify the model constructed. In general, overfitting is possible when the number of variables (i.e., radiomic features in this study) used is more than the number of data points available (i.e. the patients’ amount in this study).

The complexity degree of LASSO regression is adjusted by a control parameter λ. For the logistic regression model in this study, the LASSO method minimizes the negative loglikelihood, subjecting to the sum of the absolute value of the coefficients being less than the parameter λ. As the tuning parameter gets smaller, some coefficients shrink towards zero or are set to zero. The features with non-zero coefficients were finally selected. In this study, A value of λ= 0.001445 with log (λ) = -2.84 was selected by cross-validation via the 1-SE (standard error) criteria. The optimal tuning parameter resulted in five non-zero coefficients. The five features, CS_AD,o1_, C_a90,o7_, LRHGLE_a0,o7_, LRHGLE_a90,o7_, and LISAE, with coefficients -0.864, 1.417, -2.259, 0.100, and 0.799, respectively, were selected in the LASSO Cox regression model (Table S1).


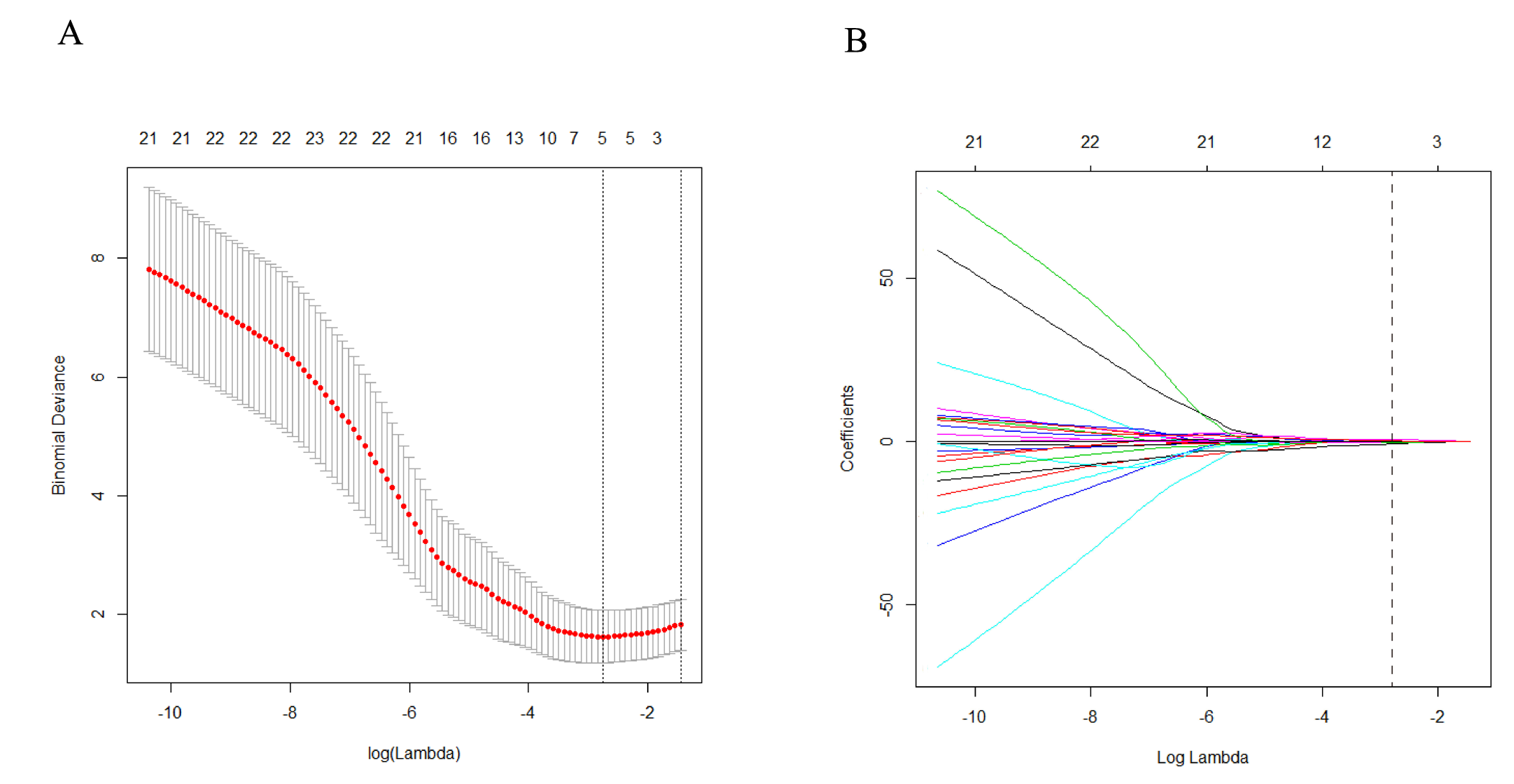


**Figure S1.** **Radiomic features selection.** (A) Radiomic features selection procedure using LASSO regression method. To determine the best features combination for building the Radscore, the control parameter λ value in the LASSO model was selected via 10-fold cross validation with minimum criteria. The x-axis is the value of log (λ) and the y-axis is the binominal deviance in the 10-fold cross-validation. The red curve indicated the average binominal deviance value with the vertical bars showing the upper and lower boundaries. The left vertical dotted line defined the λ with the least binomial deviance and was set as 0.001445 in this study. The right vertical dotted line indicates the largest value of λ such that the binominal deviance is within one standard error of the minimum binominal deviance.(B) LASSO coefficient profiles of radiomic features. A vertical line was drawn at the value selected using 10-fold cross-validation in log (λ) sequence and showed five coefficients with non-zero were indicated.

**Table S1 The coefficients of radiomic features**

| Selected Features | Features (Full name) | coefficient |
| --- | --- | --- |
|  | (Intercept) | -0.009 |
| CS_AD,o1_ | ClusterShade_AllDirection_offset1_SD | -0.864 |
| C_a90,o7_ | Correlation_angle90_offset7 | 1.417 |
| LRHGLE_a0,o7_ | LongRunHighGreyLevelEmphasis_angle0_offset7 | -2.259 |
| LRHGLE_a90,o7_ | LongRunHighGreyLevelEmphasis_angle90_offset7 | 0.100 |
| LISAE | LowIntensitySmallAreaEmphasis | 0.799 |

**Ⅲ Correlation of five features**

The correlation coefficient between each pair is less than 0.7 (Figure S2). After calculating the collinearity of the five features through the VIF function in R, the values of the five features are 1.30, 1.29, 1.21, 1.38, and 1.10 respectively, which are all less than 10, that means there is no collinearity between the five features.


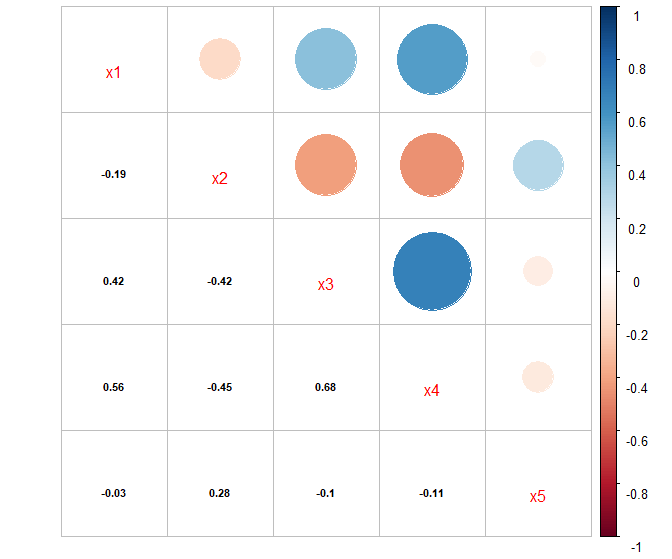


**Figure S2. Correlation of the features.** The x1-5 represents the five features: CS_AD,o1_, C_a90,o7_, LRHGLE_a0,o7_, LRHGLE_a90,o7_, and LISAE, respectively，the correlation coefficient between each pair is less than 0.7.There is no collinearity after verifying the correlation of the five features.

**IV Comparsion of Nomogram, Radscore and loculus**

The Delong’s test was performed to compare the AUCs of Nomogram, Radscore and loculus. The Nomogram had a significant improvement than the radscore and loculus alone in the training corhort (p<0.05). However, the improvement of the Nomogram model was not significant compared to radscore alone in the validation cohort (p>0.05). We speculate that this may be caused by the small sample size of the validation cohort (n=30). Detailed in Table S2 and S3.

Table S2 Comparison of ROC curves in training cohort

| Nomogram ~ Radscore | |
| --- | --- |
| Difference between areas | 0.0618 |
| Standard Error ^a^ | 0.0301 |
| 95% Confidence Interval | 0.00280 to 0.121 |
| z statistic | 2.053 |
| Significance level | P = 0.0401 |
| Nomogram ~ Loculus | |
| Difference between areas | 0.181 |
| Standard Error ^a^ | 0.0484 |
| 95% Confidence Interval | 0.0861 to 0.276 |
| z statistic | 3.738 |
| Significance level | P = 0.0002 |
| Radscore ~ Loculus | |
| Difference between areas | 0.119 |
| Standard Error ^a^ | 0.0589 |
| 95% Confidence Interval | 0.00375 to 0.235 |
| z statistic | 2.024 |
| Significance level | P = 0.0430 |

Table S3 Comparison of ROC curves in validation cohort

| Nomogram ~ Radscore | |
| --- | --- |
| Difference between areas | 0.0769 |
| Standard Error ^a^ | 0.0819 |
| 95% Confidence Interval | -0.0836 to 0.237 |
| z statistic | 0.939 |
| Significance level | P = 0.3476 |
| Nomogram ~ Loculus | |
| Difference between areas | 0.301 |
| Standard Error ^a^ | 0.100 |
| 95% Confidence Interval | 0.105 to 0.497 |
| z statistic | 3.010 |
| Significance level | P = 0.0026 |
| Radscore ~ Loculus | |
| Difference between areas | 0.378 |
| Standard Error ^a^ | 0.174 |
| 95% Confidence Interval | 0.0375 to 0.719 |
| z statistic | 2.176 |
| Significance level | P = 0.0296 |

**References**

1. Liang W, Yang P, Huang R, Xu L, Wang J, Liu W, et al. A Combined Nomogram Model to Preoperatively Predict Histologic Grade in Pancreatic Neuroendocrine Tumors. *Clinical cancer research : an official journal of the American Association for Cancer Research* (2019) 25(2):584-94. doi: 10.1158/1078-0432.CCR-18-1305. PubMed PMID: 30397175.

2. Jiang Y, Zhang Q, Hu Y, Li T, Yu J, Zhao L, et al. ImmunoScore Signature: A Prognostic and Predictive Tool in Gastric Cancer. *Annals of surgery* (2018) 267(3):504-13. doi: 10.1097/SLA.0000000000002116. PubMed PMID: 28002059.

3. Jiang Y, Chen C, Xie J, Wang W, Zha X, Lv W, et al. Radiomics signature of computed tomography imaging for prediction of survival and chemotherapeutic benefits in gastric cancer. *EBioMedicine* (2018) 36:171-82. doi: 10.1016/j.ebiom.2018.09.007. PubMed PMID: 30224313; PubMed Central PMCID: PMC6197796.
